# Bis(pyrrolidin-1-yl)phosphinic (2,4-di­fluoro­benzo­yl)amide

**DOI:** 10.1107/S1600536812034733

**Published:** 2012-08-11

**Authors:** Mojtaba Keikha, Mehrdad Pourayoubi, Jerry P. Jasinski, James A. Golen

**Affiliations:** a Department of Chemistry, Ferdowsi University of Mashhad, Mashhad, Iran; b Department of Chemistry, Keene State College, 229 Main Street, Keene, NH 03435-2001, USA

## Abstract

The P atom in the title mol­ecule, C_15_H_20_F_2_N_3_O_2_P, is in a distorted tetra­hedral P(O)(N)(N)_2_ environment. The phosphoryl group and the NH unit adopt a *syn* orientation with respect to each other. An F atom at position 2 and an H atom at position 6 are found to occupy similar sites in a 0.70:0.30 ratio and were refined with fixed occupancies. The pyrrolidin-1-yl rings are disordered over two sets of sites, with site occupancies of 0.566 (6) and 0.434 (6), and were refined using a two-part model. In the crystal, hydrogen-bonded dimers linked by pairs of N—H⋯O(P) hydrogen bonds generate an *R*_2_^2^(8) ring motif.

## Related literature

For background and related crystal structures, see: Pourayoubi *et al.* (2011 ▶, 2012 ▶). For the preparation of the starting compound, see: Pourayoubi *et al.* (2012 ▶). For graph-set motifs, see: Bernstein *et al.* (1995 ▶).
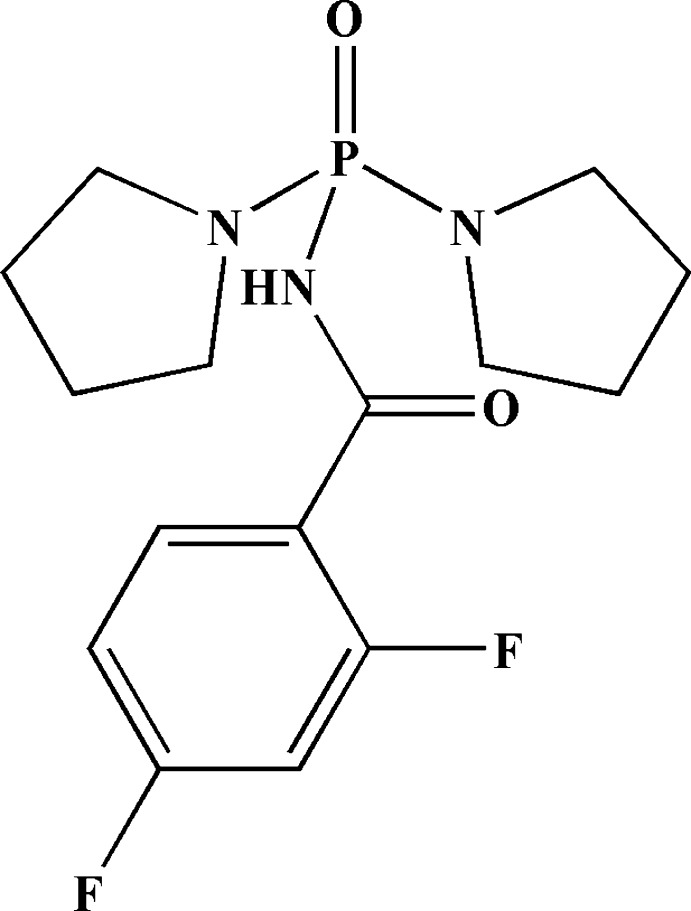


## Experimental

### 

#### Crystal data

C_15_H_20_F_2_N_3_O_2_P
*M* *_r_* = 343.31
Monoclinic, 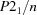

*a* = 9.1028 (3) Å
*b* = 9.9477 (2) Å
*c* = 18.5465 (5) Å
β = 92.268 (3)°
*V* = 1678.11 (8) Å^3^
*Z* = 4
Mo *K*α radiation
μ = 0.20 mm^−1^
*T* = 173 K
0.40 × 0.30 × 0.20 mm

#### Data collection

Oxford Diffraction Xcalibur Eos Gemini diffractometer
Absorption correction: multi-scan (*CrysAlis RED*; Oxford Diffraction, 2010 ▶) *T*_min_ = 0.926, *T*_max_ = 0.962
17134 measured reflections
4339 independent reflections
3828 reflections with *I* > 2σ(*I*)
*R*_int_ = 0.017

#### Refinement

*R*[*F* ^2^ > 2σ(*F* ^2^)] = 0.040
*wR*(*F* ^2^) = 0.108
*S* = 1.03
4339 reflections
300 parameters
25 restraints
H atoms treated by a mixture of independent and constrained refinement
Δρ_max_ = 0.38 e Å^−3^
Δρ_min_ = −0.30 e Å^−3^

### 

Data collection: *CrysAlis PRO* (Oxford Diffraction, 2010 ▶); cell refinement: *CrysAlis PRO*; data reduction: *CrysAlis RED* (Oxford Diffraction, 2010 ▶); program(s) used to solve structure: *SHELXS97* (Sheldrick, 2008 ▶); program(s) used to refine structure: *SHELXL97* (Sheldrick, 2008 ▶); molecular graphics: *SHELXTL* (Sheldrick, 2008 ▶); software used to prepare material for publication: *SHELXTL* and *enCIFer* (Allen *et al.*, 2004 ▶).

## Supplementary Material

Crystal structure: contains datablock(s) I, global. DOI: 10.1107/S1600536812034733/pv2575sup1.cif

Structure factors: contains datablock(s) I. DOI: 10.1107/S1600536812034733/pv2575Isup2.hkl

Supplementary material file. DOI: 10.1107/S1600536812034733/pv2575Isup3.cml

Additional supplementary materials:  crystallographic information; 3D view; checkCIF report

## Figures and Tables

**Table 1:** Hydrogen-bond geometry (Å, °)

*D*—H⋯*A*	*D*—H	H⋯*A*	*D*⋯*A*	*D*—H⋯*A*
N1—H1*N*⋯O1^i^	0.84 (1)	1.95 (1)	2.7845 (14)	170 (2)

Symmetry code: (i) 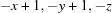.

**Fig. 1.**
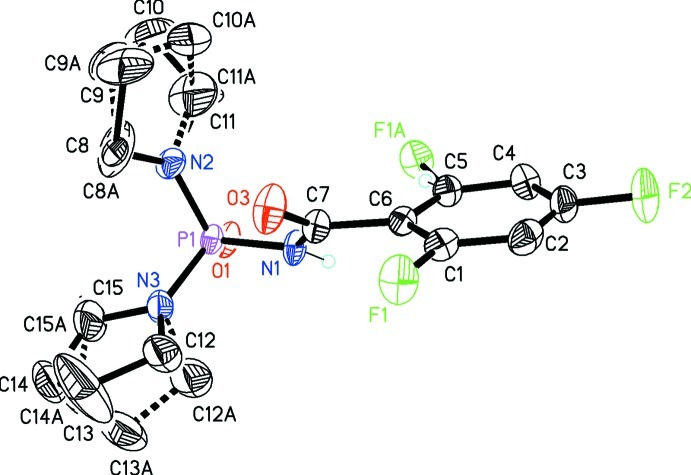
An ORTEP drawing and atom labeling scheme for the title compound. Displacement ellipsoids are given at 50% probability level. Dashed lines indicate disordered (N2, C8—C11 and N3, C12—C15) rings. H1N and H1A atoms are drawn as small spheres of arbitrary radii and other H atoms are omitted for clarity.

**Fig. 2.**
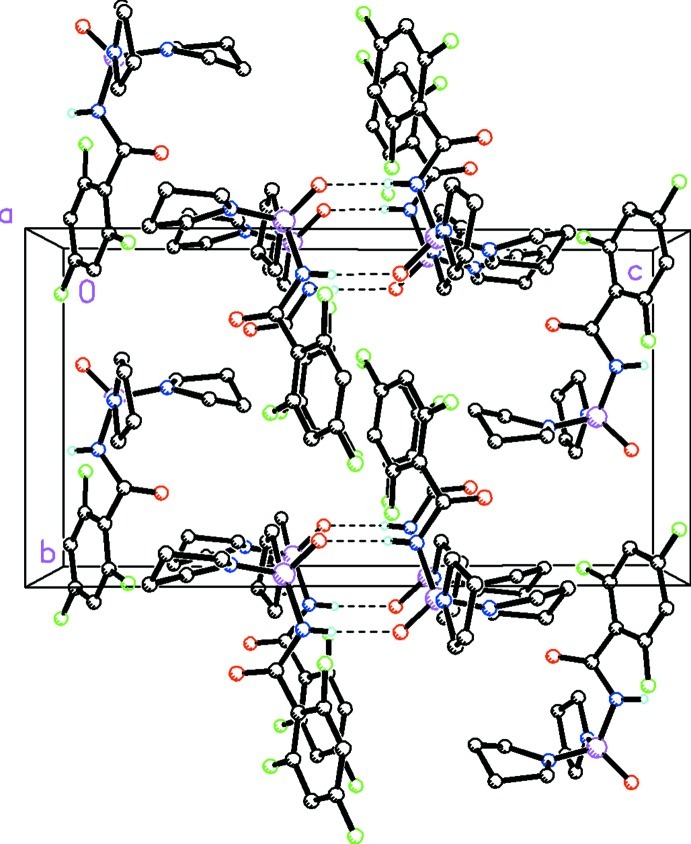
Packing diagram of the title compound viewed along the a axis. Dashed lines indicate inversion dimers linked by pairs of N—H···O(P) hydrogen bonds generating R22(8) motif rings. H atoms non-participating in hydrogen-bonding and the minor component of both disordered pyrrolidine substituents (C8A—C11A and C12A—C15A) have been removed for clarity.

## supplementary crystallographic information

### Comment

Following the previous work in our research group on phosphoric triamides (Pourayoubi *et al.*, 2011; 2012), herein, we report the synthesis and crystal structure of the title compound.

In the C(O)NHP(O) skeleton of the title phosphoric triamide (Fig. 1), the phosphoryl group adopts an *anti* orientation with respect to the carbonyl group; whereas it is in a *syn* position relative to the N—H unit. The phosphorus atom has a distorted tetrahedral configuration and the P—N bonds in the P(O)[NC_4_H_8_]_2_ fragment are shorter than the other P—N bond in the molecule. The P═O and C═O bond lengths, and P—N—C bond angles are within the expected values (Pourayoubi *et al.*, 2012). The atoms F1/H1A and F1A/H1 are found to occupy similar sites in the ratio of 70/30 and are refined with fixed occupancies. Both pyrrolidine substituents (rings N2, C8—C11 and N3, C12—C15) are disordered over two sets of sites, with site occupancies of 0.566 (6) and 0.434 (6) and are refined using a two part model.

In the crystal structure, pairs of intermolecular P═O···H—N hydrogen bonds (Table 1 and Fig. 2) form hydrogen-bonded dimers as *R*_2_^2^(8) ring (Bernstein *et al.*, 1995).

### Experimental

2,4-F_2_—C_6_H_3_C(O)NHP(O)Cl_2_ was prepared according to the literature method (Pourayoubi *et al.*, 2012). To a solution of 2,4-F_2_–C_6_H_3_C(O)NHP(O)Cl_2_ (2 mmol) in CHCl_3_ (20 ml), a solution of pyrrolidine (8 mmol) in CHCl_3_ (10 ml) was added dropwise at 273 K. After 4 h of stirring, the solvent was evaporated at room temperature and the solid was washed with distilled water. Single crystals of the title compound were obtained from a mixture of methanol/acetonitrile (1:1) after slow evaporation at room temperature.

### Refinement

Fluorine atom F1 and hydrogen atom H1A (and F1A and H1) were found to occupy similar sites in the ratio of 70/30 and were refined with fixed occupancies. Rings N2, C8—C11 and N3, C12—C15 were disordered and were refined using a two part model. Hydrogen atom H1N was found from a Fourier difference map and was refined with N—H distance of 0.87 Å and 1.20 × *U*_eq_ of N atom. All other hydrogen atoms were placed in calculated positions, CH_2_ 0.99 Å, C(Ar)—H 0.95 Å with 1.20 *U*_eq_ of the parent carbon atoms.

### Crystal data

**Table:**

C_15_H_20_F_2_N_3_O_2_P	*F*(000) = 720
*M**_r_* = 343.31	*D*_x_ = 1.359 Mg m^−^^3^
Monoclinic, *P*2_1_/*n*	Mo *K*α radiation, λ = 0.71073 Å
Hall symbol: -P 2yn	Cell parameters from 7108 reflections
*a* = 9.1028 (3) Å	θ = 3.3–32.3°
*b* = 9.9477 (2) Å	µ = 0.20 mm^−^^1^
*c* = 18.5465 (5) Å	*T* = 173 K
β = 92.268 (3)°	Block, colorless
*V* = 1678.11 (8) Å^3^	0.40 × 0.30 × 0.20 mm
*Z* = 4	

### Data collection

**Table:**

Oxford Diffraction Xcalibur Eos Gemini diffractometer	4339 independent reflections
Radiation source: Enhance (Mo) X-ray Source	3828 reflections with *I* > 2σ(*I*)
Graphite monochromator	*R*_int_ = 0.017
Detector resolution: 16.1500 pixels mm^-1^	θ_max_ = 28.7°, θ_min_ = 3.3°
ω scans	*h* = −12→7
Absorption correction: multi-scan (*CrysAlis RED*; Oxford Diffraction, 2010)	*k* = −12→13
*T*_min_ = 0.926, *T*_max_ = 0.962	*l* = −24→25
17134 measured reflections	

### Refinement

**Table:**

Refinement on *F*^2^	Primary atom site location: structure-invariant direct methods
Least-squares matrix: full	Secondary atom site location: difference Fourier map
*R*[*F*^2^ > 2σ(*F*^2^)] = 0.040	Hydrogen site location: inferred from neighbouring sites
*wR*(*F*^2^) = 0.108	H atoms treated by a mixture of independent and constrained refinement
*S* = 1.03	*w* = 1/[σ^2^(*F*_o_^2^) + (0.0515*P*)^2^ + 0.7979*P*] where *P* = (*F*_o_^2^ + 2*F*_c_^2^)/3
4339 reflections	(Δ/σ)_max_ = 0.001
300 parameters	Δρ_max_ = 0.38 e Å^−^^3^
25 restraints	Δρ_min_ = −0.30 e Å^−^^3^

### Special details

**Table:**

Experimental. IR (KBr, ν, cm^-1^): 3067, 2973, 2892, 1685, 1623, 1457, 1258, 1220, 1177, 1129, 1087, 1011, 968, 859, 811. ^1^H NMR (400.22 MHz, DMSO-d_6_, 293.9 K, TMS): 1.57 (m, 8H), 3.14 (m, 8H), 7.19 (t, 1H, Ar—H), 7.36 (t, ^3^J[(H,H),(H,F)] = 10.0 Hz, 1H, Ar—H), 7.66 (m, 1H, Ar—H), 9.26 p.p.m. (s, 1H, N—H). ^13^C NMR (100.64 MHz, DMSO-d_6_, 293.9 K, TMS): 26.38 (d, ^3^J(C,P) = 9.1 Hz, 4C), 46.34 (d, ^2^J(C,P) = 5.0 Hz, 4C), 105.03 (t, ^2^J(C,F) = 26.2 Hz, 1C, Ar—C), 112.16 (dd, ^2^J(C,F) = 21.6 Hz, ^4^J(C,F) = 3.5 Hz, 1C, Ar—C), 121.61 (m, 1C, Ar—C), 132.22 (dd, ^3^J(C,F) = 4.0 Hz and 11.1 Hz, 1C, Ar—C), 160.29 (d, ^3^J(C,F) = 13.6, ^1^J(C,F) = 252.1 Hz, 1C, Ar—C), 164.06 (d, ^3^J(C,F) = 12.1, ^1^J(C,F) = 251.1 Hz, 1C, Ar—C), 165.37 p.p.m. (s, 1C, C(O)). ^31^P{^1^H} NMR (162.01 MHz, DMSO-d_6_, 293.9 K, 85% H_3_PO_4_): 5.66 p.p.m. (*s*).
Geometry. All e.s.d.'s (except the e.s.d. in the dihedral angle between two l.s. planes) are estimated using the full covariance matrix. The cell e.s.d.'s are taken into account individually in the estimation of e.s.d.'s in distances, angles and torsion angles; correlations between e.s.d.'s in cell parameters are only used when they are defined by crystal symmetry. An approximate (isotropic) treatment of cell e.s.d.'s is used for estimating e.s.d.'s involving l.s. planes.
Refinement. Refinement of *F*^2^ against ALL reflections. The weighted *R*-factor *wR* and goodness of fit *S* are based on *F*^2^, conventional *R*-factors *R* are based on *F*, with *F* set to zero for negative *F*^2^. The threshold expression of *F*^2^ > σ(*F*^2^) is used only for calculating *R*-factors(gt) *etc*. and is not relevant to the choice of reflections for refinement. *R*-factors based on *F*^2^ are statistically about twice as large as those based on *F*, and *R*- factors based on ALL data will be even larger.

### Fractional atomic coordinates and isotropic or equivalent isotropic displacement parameters (Å^2^)

**Table:**

	*x*	*y*	*z*	*U*_iso_*/*U*_eq_	Occ. (<1)
P1	0.59836 (4)	0.47159 (3)	0.114620 (16)	0.02308 (10)	
F1	0.59877 (19)	1.01499 (17)	0.13543 (9)	0.0434 (4)	0.70
F1A	0.2448 (4)	0.6940 (3)	0.0502 (2)	0.0405 (8)	0.30
F2	0.17516 (14)	1.14989 (11)	0.00596 (7)	0.0617 (3)	
O1	0.53714 (12)	0.37387 (9)	0.06124 (5)	0.0316 (2)	
O3	0.57983 (14)	0.75048 (11)	0.18642 (6)	0.0425 (3)	
N1	0.53996 (13)	0.62346 (11)	0.08554 (6)	0.0264 (2)	
H1N	0.5064 (18)	0.6275 (17)	0.0427 (7)	0.032*	
N2	0.54212 (13)	0.44054 (13)	0.19479 (6)	0.0317 (3)	
N3	0.77636 (14)	0.47924 (12)	0.12213 (7)	0.0326 (3)	
C1	0.47337 (16)	0.98082 (14)	0.09944 (7)	0.0289 (3)	
H1A	0.571 (4)	1.003 (9)	0.117 (4)	0.035*	0.30
C2	0.38927 (18)	1.08460 (14)	0.07115 (8)	0.0351 (3)	
H2A	0.4184	1.1758	0.0770	0.042*	
C3	0.26117 (18)	1.05011 (15)	0.03401 (9)	0.0365 (3)	
C4	0.21545 (16)	0.91998 (16)	0.02352 (8)	0.0354 (3)	
H4A	0.1260	0.9000	−0.0024	0.042*	
C5	0.30424 (15)	0.81899 (14)	0.05208 (7)	0.0285 (3)	
H1	0.278 (4)	0.7283 (15)	0.0411 (18)	0.034*	0.70
C6	0.43458 (14)	0.84645 (12)	0.09091 (6)	0.0239 (2)	
C7	0.52591 (15)	0.73730 (13)	0.12577 (7)	0.0264 (3)	
C8	0.6225 (6)	0.4742 (8)	0.2636 (3)	0.0272 (17)	0.566 (6)
H8A	0.6806	0.5577	0.2591	0.033*	0.566 (6)
H8B	0.6888	0.4002	0.2796	0.033*	0.566 (6)
C9	0.4996 (10)	0.4928 (11)	0.3144 (4)	0.065 (2)	0.566 (6)
H9A	0.4666	0.5877	0.3146	0.078*	0.566 (6)
H9B	0.5314	0.4667	0.3641	0.078*	0.566 (6)
C10	0.3769 (6)	0.4013 (8)	0.2855 (2)	0.0748 (16)	0.566 (6)
H10A	0.2804	0.4304	0.3028	0.090*	0.566 (6)
H10B	0.3948	0.3069	0.3003	0.090*	0.566 (6)
C11	0.3829 (8)	0.4168 (11)	0.2035 (4)	0.0489 (19)	0.566 (6)
H11A	0.3491	0.3341	0.1781	0.059*	0.566 (6)
H11B	0.3231	0.4940	0.1858	0.059*	0.566 (6)
C8A	0.6167 (14)	0.4736 (16)	0.2641 (6)	0.063 (5)	0.434 (6)
H8AA	0.6565	0.5662	0.2632	0.076*	0.434 (6)
H8AB	0.6987	0.4103	0.2747	0.076*	0.434 (6)
C9A	0.5017 (15)	0.4612 (18)	0.3196 (8)	0.085 (5)	0.434 (6)
H9AA	0.5180	0.5274	0.3589	0.102*	0.434 (6)
H9AB	0.5003	0.3696	0.3404	0.102*	0.434 (6)
C10A	0.3614 (6)	0.4907 (8)	0.2760 (3)	0.0644 (17)	0.434 (6)
H10C	0.3506	0.5875	0.2646	0.077*	0.434 (6)
H10D	0.2731	0.4583	0.3003	0.077*	0.434 (6)
C11A	0.3941 (13)	0.4072 (19)	0.2095 (7)	0.079 (5)	0.434 (6)
H11C	0.3842	0.3099	0.2196	0.095*	0.434 (6)
H11D	0.3267	0.4313	0.1683	0.095*	0.434 (6)
C12	0.8722 (3)	0.5949 (3)	0.1329 (3)	0.0476 (10)	0.566 (6)
H12A	0.8306	0.6592	0.1673	0.057*	0.566 (6)
H12B	0.8873	0.6417	0.0866	0.057*	0.566 (6)
C13	1.0112 (5)	0.5376 (5)	0.1622 (6)	0.118 (3)	0.566 (6)
H13A	1.0949	0.5834	0.1402	0.142*	0.566 (6)
H13B	1.0191	0.5532	0.2150	0.142*	0.566 (6)
C14	1.0185 (4)	0.3994 (5)	0.1484 (3)	0.0579 (11)	0.566 (6)
H14A	1.0650	0.3506	0.1898	0.069*	0.566 (6)
H14B	1.0740	0.3810	0.1047	0.069*	0.566 (6)
C15	0.8656 (9)	0.3626 (9)	0.1378 (6)	0.065 (3)	0.566 (6)
H15A	0.8549	0.2979	0.0973	0.078*	0.566 (6)
H15B	0.8314	0.3181	0.1818	0.078*	0.566 (6)
C12A	0.8614 (5)	0.5743 (6)	0.0805 (4)	0.0635 (17)	0.434 (6)
H12C	0.8512	0.6670	0.0991	0.076*	0.434 (6)
H12D	0.8299	0.5725	0.0288	0.076*	0.434 (6)
C13A	1.0174 (6)	0.5242 (7)	0.0916 (6)	0.081 (2)	0.434 (6)
H13C	1.0757	0.5418	0.0487	0.098*	0.434 (6)
H13D	1.0668	0.5667	0.1343	0.098*	0.434 (6)
C14A	0.9971 (6)	0.3782 (7)	0.1029 (6)	0.082 (2)	0.434 (6)
H14C	1.0803	0.3433	0.1335	0.099*	0.434 (6)
H14D	0.9982	0.3316	0.0558	0.099*	0.434 (6)
C15A	0.8561 (9)	0.3470 (9)	0.1381 (4)	0.0336 (18)	0.434 (6)
H15C	0.8710	0.3310	0.1906	0.040*	0.434 (6)
H15D	0.8046	0.2694	0.1153	0.040*	0.434 (6)

### Atomic displacement parameters (Å^2^)

**Table:**

	*U*^11^	*U*^22^	*U*^33^	*U*^12^	*U*^13^	*U*^23^
P1	0.02981 (18)	0.02096 (16)	0.01801 (16)	0.00199 (12)	−0.00475 (11)	0.00008 (11)
F1	0.0477 (8)	0.0307 (7)	0.0504 (10)	−0.0092 (6)	−0.0175 (6)	−0.0046 (6)
F1A	0.0350 (18)	0.0305 (16)	0.055 (2)	−0.0053 (12)	−0.0065 (14)	−0.0031 (14)
F2	0.0686 (7)	0.0373 (6)	0.0781 (8)	0.0235 (5)	−0.0103 (6)	0.0113 (5)
O1	0.0503 (6)	0.0211 (4)	0.0227 (4)	0.0007 (4)	−0.0091 (4)	−0.0013 (3)
O3	0.0668 (8)	0.0314 (5)	0.0276 (5)	0.0033 (5)	−0.0184 (5)	−0.0070 (4)
N1	0.0376 (6)	0.0214 (5)	0.0193 (5)	0.0052 (4)	−0.0090 (4)	−0.0025 (4)
N2	0.0337 (6)	0.0406 (7)	0.0205 (5)	−0.0028 (5)	−0.0027 (4)	0.0009 (5)
N3	0.0317 (6)	0.0307 (6)	0.0349 (6)	0.0038 (5)	−0.0034 (5)	0.0060 (5)
C1	0.0343 (7)	0.0247 (6)	0.0277 (6)	−0.0036 (5)	0.0024 (5)	−0.0037 (5)
C2	0.0492 (9)	0.0203 (6)	0.0365 (7)	−0.0001 (6)	0.0090 (6)	0.0004 (5)
C3	0.0420 (8)	0.0285 (7)	0.0393 (8)	0.0126 (6)	0.0042 (6)	0.0053 (6)
C4	0.0319 (7)	0.0350 (7)	0.0388 (8)	0.0057 (6)	−0.0031 (6)	0.0000 (6)
C5	0.0308 (7)	0.0235 (6)	0.0310 (6)	0.0001 (5)	−0.0001 (5)	−0.0020 (5)
C6	0.0300 (6)	0.0206 (6)	0.0213 (5)	0.0015 (5)	0.0021 (5)	−0.0020 (4)
C7	0.0334 (7)	0.0222 (6)	0.0232 (6)	0.0002 (5)	−0.0038 (5)	−0.0023 (5)
C8	0.031 (2)	0.040 (5)	0.011 (3)	0.004 (2)	−0.002 (2)	0.001 (3)
C9	0.073 (5)	0.094 (4)	0.029 (3)	−0.020 (3)	0.015 (3)	−0.026 (3)
C10	0.073 (3)	0.108 (5)	0.045 (2)	−0.016 (3)	0.023 (2)	0.007 (3)
C11	0.027 (2)	0.085 (5)	0.035 (3)	−0.010 (2)	0.014 (2)	0.001 (3)
C8A	0.096 (8)	0.054 (9)	0.038 (7)	−0.004 (6)	−0.025 (6)	0.005 (6)
C9A	0.073 (7)	0.150 (12)	0.033 (4)	0.011 (6)	0.008 (4)	0.025 (6)
C10A	0.057 (3)	0.091 (5)	0.047 (3)	0.014 (3)	0.020 (2)	0.007 (3)
C11A	0.065 (6)	0.134 (12)	0.037 (4)	−0.022 (6)	−0.017 (4)	−0.003 (5)
C12	0.0330 (15)	0.0340 (15)	0.076 (3)	−0.0070 (11)	−0.0001 (15)	0.0075 (16)
C13	0.038 (2)	0.063 (3)	0.249 (10)	−0.003 (2)	−0.038 (4)	−0.015 (4)
C14	0.0310 (16)	0.061 (2)	0.080 (3)	0.0046 (15)	−0.0115 (18)	0.014 (2)
C15	0.048 (4)	0.052 (4)	0.095 (5)	0.010 (3)	0.002 (3)	0.036 (3)
C12A	0.043 (2)	0.053 (3)	0.095 (5)	−0.009 (2)	0.004 (3)	0.019 (3)
C13A	0.031 (2)	0.074 (4)	0.139 (7)	−0.005 (2)	0.009 (3)	0.024 (4)
C14A	0.030 (2)	0.069 (4)	0.148 (8)	0.013 (2)	0.000 (4)	−0.003 (5)
C15A	0.029 (3)	0.033 (3)	0.037 (3)	0.015 (2)	−0.014 (2)	−0.009 (3)

### Geometric parameters (Å, º)

**Table:**

P1—O1	1.4805 (10)	C10—H10A	0.9900
P1—N2	1.6213 (12)	C10—H10B	0.9900
P1—N3	1.6226 (13)	C11—H11A	0.9900
P1—N1	1.6832 (11)	C11—H11B	0.9900
F1—C1	1.3431 (19)	C8A—C9A	1.502 (14)
F1—H1A	0.43 (5)	C8A—H8AA	0.9900
F1A—C5	1.356 (3)	C8A—H8AB	0.9900
F1A—H1	0.49 (2)	C9A—C10A	1.513 (14)
F2—C3	1.3552 (17)	C9A—H9AA	0.9900
O3—C7	1.2165 (16)	C9A—H9AB	0.9900
N1—C7	1.3649 (16)	C10A—C11A	1.526 (13)
N1—H1N	0.841 (13)	C10A—H10C	0.9900
N2—C11A	1.425 (11)	C10A—H10D	0.9900
N2—C8A	1.467 (11)	C11A—H11C	0.9900
N2—C11	1.484 (6)	C11A—H11D	0.9900
N2—C8	1.484 (6)	C12—C13	1.472 (5)
N3—C15	1.440 (9)	C12—H12A	0.9900
N3—C12	1.453 (3)	C12—H12B	0.9900
N3—C12A	1.462 (5)	C13—C14	1.400 (7)
N3—C15A	1.526 (8)	C13—H13A	0.9900
C1—C2	1.377 (2)	C13—H13B	0.9900
C1—C6	1.3900 (18)	C14—C15	1.445 (9)
C1—H1A	0.957 (10)	C14—H14A	0.9900
C2—C3	1.374 (2)	C14—H14B	0.9900
C2—H2A	0.9500	C15—H15A	0.9900
C3—C4	1.371 (2)	C15—H15B	0.9900
C4—C5	1.3817 (19)	C12A—C13A	1.512 (7)
C4—H4A	0.9500	C12A—H12C	0.9900
C5—C6	1.3905 (18)	C12A—H12D	0.9900
C5—H1	0.953 (10)	C13A—C14A	1.480 (8)
C6—C7	1.4985 (17)	C13A—H13C	0.9900
C8—C9	1.503 (8)	C13A—H13D	0.9900
C8—H8A	0.9900	C14A—C15A	1.495 (10)
C8—H8B	0.9900	C14A—H14C	0.9900
C9—C10	1.521 (9)	C14A—H14D	0.9900
C9—H9A	0.9900	C15A—H15C	0.9900
C9—H9B	0.9900	C15A—H15D	0.9900
C10—C11	1.532 (9)		
			
O1—P1—N2	111.35 (6)	H11A—C11—H11B	109.3
O1—P1—N3	115.87 (7)	N2—C8A—C9A	105.7 (10)
N2—P1—N3	106.30 (6)	N2—C8A—H8AA	110.6
O1—P1—N1	105.63 (5)	C9A—C8A—H8AA	110.6
N2—P1—N1	110.96 (6)	N2—C8A—H8AB	110.6
N3—P1—N1	106.68 (6)	C9A—C8A—H8AB	110.6
C7—N1—P1	127.22 (9)	H8AA—C8A—H8AB	108.7
C7—N1—H1N	116.0 (12)	C8A—C9A—C10A	102.4 (10)
P1—N1—H1N	116.3 (12)	C8A—C9A—H9AA	111.3
C11A—N2—C8A	107.0 (7)	C10A—C9A—H9AA	111.3
C8A—N2—C11	111.2 (6)	C8A—C9A—H9AB	111.3
C11A—N2—C8	109.0 (6)	C10A—C9A—H9AB	111.3
C11—N2—C8	113.1 (4)	H9AA—C9A—H9AB	109.2
C11A—N2—P1	123.8 (6)	C9A—C10A—C11A	98.0 (9)
C8A—N2—P1	127.5 (5)	C9A—C10A—H10C	112.2
C11—N2—P1	118.4 (3)	C11A—C10A—H10C	112.2
C8—N2—P1	125.6 (2)	C9A—C10A—H10D	112.2
C15—N3—C12	106.2 (4)	C11A—C10A—H10D	112.2
C15—N3—C12A	108.8 (4)	H10C—C10A—H10D	109.8
C12—N3—C15A	112.2 (3)	N2—C11A—C10A	104.1 (9)
C12A—N3—C15A	113.7 (4)	N2—C11A—H11C	110.9
C15—N3—P1	122.2 (4)	C10A—C11A—H11C	110.9
C12—N3—P1	129.90 (15)	N2—C11A—H11D	110.9
C12A—N3—P1	122.2 (2)	C10A—C11A—H11D	110.9
C15A—N3—P1	116.2 (3)	H11C—C11A—H11D	108.9
F1—C1—C2	116.64 (14)	N3—C12—C13	104.3 (3)
F1—C1—C6	120.47 (14)	N3—C12—H12A	110.9
C2—C1—C6	122.88 (13)	C13—C12—H12A	110.9
C2—C1—H1A	117 (5)	N3—C12—H12B	110.9
C6—C1—H1A	119 (5)	C13—C12—H12B	110.9
C3—C2—C1	116.86 (13)	H12A—C12—H12B	108.9
C3—C2—H2A	121.6	C14—C13—C12	111.0 (4)
C1—C2—H2A	121.6	C14—C13—H13A	109.4
F2—C3—C4	118.00 (15)	C12—C13—H13A	109.4
F2—C3—C2	118.39 (14)	C14—C13—H13B	109.4
C4—C3—C2	123.62 (13)	C12—C13—H13B	109.4
C3—C4—C5	117.54 (14)	H13A—C13—H13B	108.0
C3—C4—H4A	121.2	C13—C14—C15	102.8 (5)
C5—C4—H4A	121.2	C13—C14—H14A	111.2
F1A—C5—C4	115.48 (19)	C15—C14—H14A	111.2
F1A—C5—C6	121.66 (19)	C13—C14—H14B	111.2
C4—C5—C6	122.00 (13)	C15—C14—H14B	111.2
C4—C5—H1	118 (2)	H14A—C14—H14B	109.1
C6—C5—H1	120 (2)	N3—C15—C14	110.8 (6)
C1—C6—C5	117.10 (12)	N3—C15—H15A	109.5
C1—C6—C7	120.90 (12)	C14—C15—H15A	109.5
C5—C6—C7	121.91 (11)	N3—C15—H15B	109.5
O3—C7—N1	123.49 (12)	C14—C15—H15B	109.5
O3—C7—C6	121.16 (12)	H15A—C15—H15B	108.1
N1—C7—C6	115.32 (11)	N3—C12A—C13A	103.2 (4)
N2—C8—C9	102.3 (5)	N3—C12A—H12C	111.1
N2—C8—H8A	111.3	C13A—C12A—H12C	111.1
C9—C8—H8A	111.3	N3—C12A—H12D	111.1
N2—C8—H8B	111.3	C13A—C12A—H12D	111.1
C9—C8—H8B	111.3	H12C—C12A—H12D	109.1
H8A—C8—H8B	109.2	C14A—C13A—C12A	102.8 (4)
C8—C9—C10	105.1 (6)	C14A—C13A—H13C	111.2
C8—C9—H9A	110.7	C12A—C13A—H13C	111.2
C10—C9—H9A	110.7	C14A—C13A—H13D	111.2
C8—C9—H9B	110.7	C12A—C13A—H13D	111.2
C10—C9—H9B	110.7	H13C—C13A—H13D	109.1
H9A—C9—H9B	108.8	C13A—C14A—C15A	112.3 (5)
C9—C10—C11	103.6 (5)	C13A—C14A—H14C	109.1
C9—C10—H10A	111.0	C15A—C14A—H14C	109.1
C11—C10—H10A	111.0	C13A—C14A—H14D	109.1
C9—C10—H10B	111.0	C15A—C14A—H14D	109.1
C11—C10—H10B	111.0	H14C—C14A—H14D	107.9
H10A—C10—H10B	109.0	C14A—C15A—N3	98.4 (6)
N2—C11—C10	101.4 (6)	C14A—C15A—H15C	112.1
N2—C11—H11A	111.5	N3—C15A—H15C	112.1
C10—C11—H11A	111.5	C14A—C15A—H15D	112.1
N2—C11—H11B	111.5	N3—C15A—H15D	112.1
C10—C11—H11B	111.5	H15C—C15A—H15D	109.7
			
O1—P1—N1—C7	157.09 (12)	C5—C6—C7—O3	−137.01 (15)
N2—P1—N1—C7	36.30 (14)	C1—C6—C7—N1	−142.04 (13)
N3—P1—N1—C7	−79.07 (13)	C5—C6—C7—N1	41.38 (18)
O1—P1—N2—C11A	−43.6 (9)	C11A—N2—C8—C9	−14.5 (11)
N3—P1—N2—C11A	−170.6 (9)	C8A—N2—C8—C9	−8 (27)
N1—P1—N2—C11A	73.7 (9)	C11—N2—C8—C9	−9.0 (9)
O1—P1—N2—C8A	153.3 (8)	P1—N2—C8—C9	151.5 (5)
N3—P1—N2—C8A	26.3 (8)	N2—C8—C9—C10	29.3 (9)
N1—P1—N2—C8A	−89.3 (8)	C8—C9—C10—C11	−39.2 (10)
O1—P1—N2—C11	−48.0 (5)	C11A—N2—C11—C10	37 (7)
N3—P1—N2—C11	−175.0 (5)	C8A—N2—C11—C10	−14.5 (10)
N1—P1—N2—C11	69.4 (5)	C8—N2—C11—C10	−14.4 (8)
O1—P1—N2—C8	152.4 (4)	P1—N2—C11—C10	−176.5 (4)
N3—P1—N2—C8	25.4 (4)	C9—C10—C11—N2	31.8 (9)
N1—P1—N2—C8	−90.2 (4)	C11A—N2—C8A—C9A	−1.6 (15)
O1—P1—N3—C15	−55.4 (5)	C11—N2—C8A—C9A	3.8 (13)
N2—P1—N3—C15	68.9 (5)	C8—N2—C8A—C9A	−175 (28)
N1—P1—N3—C15	−172.6 (5)	P1—N2—C8A—C9A	163.7 (8)
O1—P1—N3—C12	141.4 (3)	N2—C8A—C9A—C10A	−27.4 (14)
N2—P1—N3—C12	−94.3 (3)	C8A—C9A—C10A—C11A	43.4 (14)
N1—P1—N3—C12	24.2 (3)	C8A—N2—C11A—C10A	30.0 (14)
O1—P1—N3—C12A	92.4 (4)	C11—N2—C11A—C10A	−100 (8)
N2—P1—N3—C12A	−143.3 (4)	C8—N2—C11A—C10A	30.2 (13)
N1—P1—N3—C12A	−24.8 (4)	P1—N2—C11A—C10A	−136.0 (7)
O1—P1—N3—C15A	−54.8 (3)	C9A—C10A—C11A—N2	−45.7 (14)
N2—P1—N3—C15A	69.5 (3)	C15—N3—C12—C13	−5.9 (7)
N1—P1—N3—C15A	−172.0 (3)	C12A—N3—C12—C13	−105.9 (6)
F1—C1—C2—C3	179.74 (15)	C15A—N3—C12—C13	−5.0 (6)
C6—C1—C2—C3	0.9 (2)	P1—N3—C12—C13	159.3 (5)
C1—C2—C3—F2	179.47 (13)	N3—C12—C13—C14	18.5 (8)
C1—C2—C3—C4	−0.7 (2)	C12—C13—C14—C15	−22.6 (9)
F2—C3—C4—C5	179.73 (14)	C12—N3—C15—C14	−7.8 (8)
C2—C3—C4—C5	−0.1 (2)	C12A—N3—C15—C14	34.0 (8)
C3—C4—C5—F1A	170.3 (2)	P1—N3—C15—C14	−174.4 (4)
C3—C4—C5—C6	0.8 (2)	C13—C14—C15—N3	18.6 (9)
F1—C1—C6—C5	−179.07 (14)	C15—N3—C12A—C13A	−15.8 (7)
C2—C1—C6—C5	−0.2 (2)	C12—N3—C12A—C13A	77.2 (6)
F1—C1—C6—C7	4.2 (2)	C15A—N3—C12A—C13A	−19.4 (7)
C2—C1—C6—C7	−176.98 (13)	P1—N3—C12A—C13A	−167.4 (4)
F1A—C5—C6—C1	−169.5 (2)	N3—C12A—C13A—C14A	28.8 (8)
C4—C5—C6—C1	−0.6 (2)	C12A—C13A—C14A—C15A	−30.9 (10)
F1A—C5—C6—C7	7.2 (3)	C13A—C14A—C15A—N3	18.5 (8)
C4—C5—C6—C7	176.09 (13)	C15—N3—C15A—C14A	−33 (6)
P1—N1—C7—O3	14.1 (2)	C12—N3—C15A—C14A	−42.0 (5)
P1—N1—C7—C6	−164.21 (10)	C12A—N3—C15A—C14A	1.4 (6)
C1—C6—C7—O3	39.6 (2)	P1—N3—C15A—C14A	151.4 (4)

### Hydrogen-bond geometry (Å, º)

**Table:**

*D*—H···*A*	*D*—H	H···*A*	*D*···*A*	*D*—H···*A*
N1—H1*N*···O1^i^	0.84 (1)	1.95 (1)	2.7845 (14)	170 (2)

Symmetry code: (i) −*x*+1, −*y*+1, −*z*.
